# Evaluation of Type-Specific Real-Time PCR Assays Using the LightCycler and J.B.A.I.D.S. for Detection of Adenoviruses in Species HAdV-C

**DOI:** 10.1371/journal.pone.0026862

**Published:** 2011-10-27

**Authors:** Morris S. Jones, Nolan Ryan Hudson, Carl Gibbins, Stephen L. Fischer

**Affiliations:** 1 Viral and Rickettsial Disease Laboratory, California Department of Health Services, Richmond, California, United States of America; 2 Clinical Investigation Facility, David Grant Medical Center, Travis Air Force Base, California, United States of America; 3 Naval Hospital Camp Pendleton, Camp Pendleton, California, United States of America; University of Kansas Medical Center, United States of America

## Abstract

Sporadically, HAdVs from species HAdV-C are detected in acute respiratory disease outbreaks. To rapidly type these viruses, we designed real-time PCR assays that detect and discriminate between adenovirus types HAdV-C1, -C2, -C5, and -C6. Sixteen clinical isolates from the California Department of Public Health were used to validate the new assays. Type-specific TaqMan real-time PCR assays were designed and used independently to successfully identify 16 representative specimens. The lower limit of detection for our LightCycler singleplex real-time PCR assays were calculated to be 100, 100, 100, and 50 genomic copies per reaction for HAdV-C1, HAdV-C2, HAdV-C5 and HAdV-C6, respectively. The results for the singleplex J.B.A.I.D.S. assays were similar. Our assays did not cross-react with other adenoviruses outside of species HAdV-C, respiratory syncytial virus, influenza, or respiratory disease causing bacteria. These assays have the potential to be useful as diagnostic tools for species HAdV-C infection.

## Introduction

Human adenoviruses (HAdVs) were the first respiratory viruses to be isolated and characterized. Epidemiological studies show that adenoviruses are a common cause of epidemic respiratory illness in crowded adult populations [1], [2]. Human adenoviruses are members of the genus *Mastadenovirus* and are grouped into seven species (A–G), based on their nucleic acid characteristics, penton, hexon, and fiber protein characteristics, biological properties and phylogenetic analysis [3], [4], [5], [6], [7]. They are associated with a broad range of symptoms, including acute respiratory disease (ARD), gastroenteritis, keratoconjunctivitis, and genitourinary infections [1], [2].

Viruses in species HAdV-C are weakly pathogenic in adult populations [8]. In contrast, when they infect children under 2 years of age, the clinical outcome can lead to acute respiratory disease, intussusception [9], [10], pneumonia, or myocarditis in infants under age 1 [11]–[12]. Moreover, an association between prenatal species HAdV-C infection and development of childhood acute leukemia was recently reported [13].

In the previous century, HAdVs were detected by tissue culture and discriminated by type-specific serum neutralization methods [14]. However, traditional (probeless) PCR assays have since replaced these methods owing to their greater speed, significantly lower cost, and to the decline in availability of type-specific antisera [15]. A variety of reliable PCR assays have been developed and used, including species-specific [16] and type-specific [17] tests. Universal PCR assays paired with sequence analysis has been used to provide a truly comprehensive detection and discrimination method for all HAdV types [18]. Real-time (probe-based) PCR platforms now offer even greater efficiency, improved sensitivity and specificity, and the added information value resulting from quantitative analysis of viral titers [19], [20], [21].

In this study we developed a series of real-time PCR assays for both the LightCycler and the military Joint Biological Agent Identification Detection System (J.B.A.I.D.S.) platforms which can detect and discriminate between HAdVs in species HAdV-C. Combined, these tools offer a rapid, high-throughput method for detection and discrimination of viruses in species HAdV-C. These assays will allow for much more rapid outbreak assessment, and, if validated as in-house diagnostic assays, more rapid individual and public health responses.

## Results

### LightCycler and J.B.A.I.D.S. TaqMan real-time PCR assays

The detection of Human adenoviruses C1 (HAdV-C1), -C2, -C5, and –C6 by real-time PCR from cultured isolates is summarized in Table 1. All samples were tested on the LightCycler 2.0 and the J.B.A.I.D.S platforms. All positive samples were correctly identified (Table 1).

**Table 1:** Summary of Human adenovirus (HAdV) detection by real-time PCR using the LightCycler 2.0 and J.B.A.I.D.S. in cultured isolates from the CDPH.

Samples	LightCycler 2.0 real-time PCR		J.B.A.I.D.S. real-time PCR	
	Positive	Negative	Positive	Negative
HAdV-C1	6	0	6	0
HAdV-C2	4	0	4	0
HAdV-C5	5	0	5	0
HAdV-C6	1	0	1	0

### Lower limit of detection for singleplex assays

**Figure 1:**
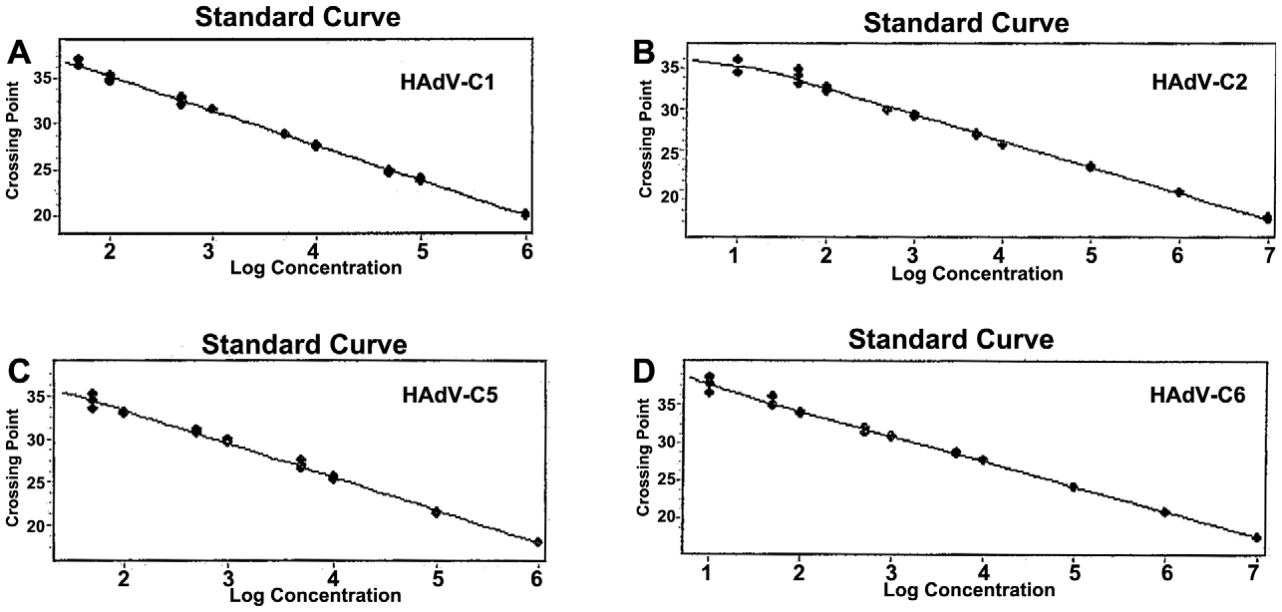
Standard curves for amplification of HAdV-C1, -C2, -C5, and -C6. Detection by real-time LightCycler PCR in a series of dilutions of genomic HAdV-C1, -C2, -C5, and -C6 DNA. Linear regression of the standard curves for (A) HAdV- C1, (B) HAdV-C2, (C) HAdV-C5, and (D) HAdV-C6. We defined the LLOD as the last dilution before the crossing points ceased to increase.

Standard curves were created using different dilutions of genomic DNA ranging from 50 to 10^7^ genome copies per assay from each HAdV (Fig. 1). Using the LightCycler 2.0, the lower limit of detection (LLOD) for our singleplex assays was 100, 100, 100, and 50 genomic copies per reaction for HAdV-C1, -C2, -C5, and –C6, respectively (Table 2). Using the J.B.A.I.D.S. the LLOD for our singleplex assays was 50, 50, 100, and 100 genomic copies per reaction for HAdV-C1, -C2, -C5, and –C6, respectively (Table 2). We defined the LLOD as the last dilution before the crossing points stopped increasing. The singleplex assay results for HAdV-C1, -C2, -C5, and –C6 were similar on the J.B.A.I.D.S. system (Table 2). Linear regression of the *CP* values and the quantity of genomic DNA revealed negative linearity for all curves (Fig. 1), corresponding to 100, 97, 93, and 99% PCR efficiency for HAdV-C1, -C2, -C5, and –C6 singleplex assays, respectively. The dynamic range for the HAdV-C2 and –C6 assays was 50 to 1.0×10^7^ DNA genomic copies per PCR reaction and 10^2^ to 1.0×10^6^ DNA genomic copies per PCR reaction for HAdV-C1 and HAdV-C5. These experiments were repeated twice with similar results.

**Table 2:** LLOD for HAdV singleplex reactions (genome copies/reaction).

	LLOD LightCycler 2.0	LLOD J.B.A.I.D.S.
HAdV-C1	100	50
HAdV-C2	100	50
HAdV-C5	100	100
HAdV-C6	50	100

### Specificity of Real-Time PCR assays

Because adenoviruses in species HAdV-C are well conserved in the hexon and fiber genes, we wanted to determine if our assays were specific to the target HAdV types. Analytical specificity data is shown in Table 3. Assays that detect HAdV-C2, -C5, and –C6 showed low level amplification with HAdV-C1, -C2, and –C5, respectively after 35 cycles. However, our assays did not react with HAdV-B3, -E4, -B7, -B11, -B14, or –B21. In addition, our real-time PCR assays did not generate false positive results when challenged with genomic DNA extracted from other agents which cause respiratory disease such as *Haemophilus influenza*, *Influenza A virus*, *Human rhinovirus*, *Human parainfluenza virus*, *Human respiratory syncytial virus*, *Chlamydophila pneumonia*, *Escherichia Coli*, *Klebsiella pneumonia*, *Pseudomonas aeruginosa*, *Mycoplasma pneumonia*, and *Legionella Pneumophila*.

**Table 3:** Specificity table of each HAdV assay.

Pathogen	HAdV-C1	HAdV-C2	HAdV-C5	HAdV-C6
HAdV-C1	+	a		
HAdV-C2		+	b	
HAdV-C5			+	c
HAdV-C6				+
HAdV-B3				
HAdV-E4				
HAdV-B7				
HAdV-B11				
HAdV-B14				
HAdV-B21				
Haemophilus influenzae				
Influenza A virus				
Human rhinovirus				
Human parainfluenza virus				
Human respiratory syncytial virus				
*Chlamydophila pneumoniae*				
*Escherichia Coli*				
*Klebsiella pneumonia*				
*Pseudomonas aeruginosa*				
*Mycoplasma pneumoniae*				
*Legionella Pneumophila*				

a – Low levels of HAdV-C1 was detected with the HAdV-C2 primers and probes after 35 cycles.

b – Low levels of HAdV-C2 was detected with the HAdV-C5 primers and probes after 35 cycles.

c – Low levels of HAdV-C5 was detected with the HAdV-C6 primers and probes after 35 cycles.

## Discussion

There has been a need for a rapid, accurate diagnostic type specific assays which are able to differentiate HAdVs. Currently there are type specific real-time PCR assays for species HAdV-B and HAdV-E [20], [21], but not for species HAdV-C. Since viruses in species HAdV-C cause morbidity in people who are immunosupressed and/or immunocompromised [22], [23], it is critical to be able to detect and discriminate between all common respiratory HAdV types. We describe the first series of real-time PCR assays which can discriminate between the four viruses which are known in species HAdV-C.

Since it is not possible to predict outbreaks, for example the HAdV-B14 outbreak in 2006 [24], [25], it is important to have high-quality assays readily available that can determine which adenovirus is present. Although the described singleplex assays can be used to determine whether or not nucleic acid from a virus in species HAdV-C is present in less than two hours, which is faster than conventional PCR, we recommend that these assays be used on isolates that are untypeable via PCR assays that have been validated for clinical samples [21].

In terms of cost, the described real-time PCR assays are less labor intensive which directly decreases the cost of testing each sample. Moreover, the cost of the primers and probes is less than a dollar per test. Thus, our assays are rapid and inexpensive.

One limitation to the described assays is that they target the hexon gene which has been shown to be incomplete when typing HAdVs [3]. If a HAdV which has the fiber of HAdV-C1 and the hexon of HAdV-C5, using the singleplex assays described in this study, the virus would be incorrectly typed as HAdV-C5. However, if these assays are used on a sample that contains two viruses from species HAdV-C, both viruses will be detected in separate capillaries.

**Figure 2:**
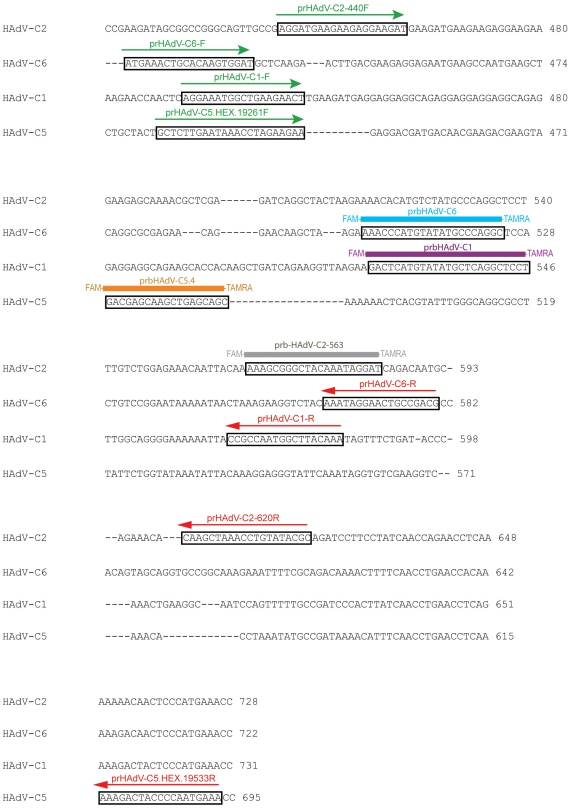
Primer design for type specific primer/probe pairs in species HAdV-C hexon.

The described singleplex assays were able to accurately detect the four known viruses in species HAdV-C. Unfortunately, after 35 cycles the primer/probe pairs for HAdV-C2, -C5, and –C6 amplified low levels of HAdV-C1, -C2, and –C5, respectively. This may be due to the fact that the viruses in species HAdV-C are genetically similar [26]. However, this was surprising in light of the genetic diversity that is present in the hexon genes of the viruses in species HAdV-C (Fig. 2). This was the region we used to design all primer/probe pairs. This demonstrates the complexity of designing type-specific assays using real-time PCR.

### Conclusions

We generated single real-time PCR assays that are sensitive and specific and provide accurate quantitation of HAdV in species HAdV-C. These assays have the potential to be useful as routine diagnostic tools for the rapid detection of clinical samples on already existing platforms which are positive for HAdV-C1, -C2, -C5, and –C6.

## Materials and Methods

### Ethics Statement

The work reported herein was approved by the Institutional Review Board at the David Grant USAF Medical Center. Informed Consent was not required, because we did not use clinical samples.

### Viral Isolates

Samples collected at CDPH were clinical isolates cultured in A549 cells, and subsequently frozen at −80°C and transported on dry ice for testing. Aliquots used in this study were subjected to one freeze/thaw cycle in the process of aliquotting upon receipt at CDPH, and one further freeze/thaw cycle when aliquotted for shipment to the David Grant Medical Center. The A549 cells used in this study were used in a previous study [5], [20] and are a common cell line used for adenovirus research.

### Processing of cultured viruses for PCR

Cultured isolates were processed as follows. Nucleic acid was extracted from 175 µL aliquots using the MagNA Pure LC DNA Isolation Kit I (Roche, Indianapolis, IN, USA) according to the manufacturers' recommendations for the MagNA Pure LC automated nucleic acid extraction system. Adenovirus strains HAdV-B14 (VR-15), -B3 (VR-3), -E4 (VR-1572), -B7 (VR-7), -B11 (VR-12), -B21 (VR-256), Haemophilus influenza, Influenza A virus (ATCC VR-96), Human rhinovirus 14 (ATCC VR-284), Human parainfluenza virus 2 (ATCC VR-92), Human respiratory syncytial virus (ATCC VR-26), *Chlamydophila pneumonia* (ATCC 53592), *Escherichia Coli*, *Klebsiella pneumonia* (ATCC 13883), *Pseudomonas aeruginosa* (ATCC 97), *Mycoplasma pneumonia*, and *Legionella Pneumophila* (ATCC 33152) were acquired from the American Type Culture Collection (ATCC; Manassas, VA, USA). Genomic DNA from each adenovirus strain was quanitated by calculating the number of genomes based on the A260 reading on the NanaDrop 8000 (Thermo Scientific, Wilmington).

### Optimization of assays

First, we performed experiments to determine the appropriate annealing temperature for each series of primers via conventional PCR. Reaction conditions were as follows: initial denaturation at 95°C for 15 min, followed by 40 cycles of denaturation at 95°C for 15 s, annealing temperature gradient ranged from 48°C to 65°C for 30 s, and extension at 72°C for 30 s. Each singleplex assay was optimized for MgCl_2_ concentration as well as extension and annealing times. To optimize the MgCl_2_ concentration, we used 0, 1, 2, 3, 4, 5 and 6 mM MgCl_2_ as the final concentration of the PCR reaction. We also amplified a fixed amount of genomic DNA with different annealing (25, 20, and 15s) and extension times (20, 15, 10, and 5s). The FastStart High Fidelity PCR System kit was used for all conventional PCR (Roche).

### Quantitative real-time PCR

The primers used to detect the different viruses in species HAdV-C are listed in Table 4. Experiments were performed on a Joint Biological Agent Identification and Diagnostic System (J.B.A.I.D.S) and the LightCycler 2.0 (Roche). All J.B.A.I.D.S. experiments were performed using the same primers, probes, and conditions as the LightCycler 2.0. The J.B.A.I.D.S. thermocycler was conceived and developed to rapidly identify biological warfare agents and other pathogens of concern for the U.S. Military. For real-time J.B.A.I.D.S. PCR, cycling was carried out in a J.B.A.I.D.S. real-time thermocycler (Idaho Technologies, UT, USA) using 1 µl of extracted DNA in 2 µls of LC FastStart DNA Master HybProbe mix (Roche, Indianapolis, IN, USA) (Roche), containing 5 mM MgCl_2_ and 400 nM forward and reverse primers. Reaction conditions were as follows: initial denaturation at 95°C for 15 min, followed by 45 cycles of denaturation at 95°C for 1 s, annealing at 60°C for 15 s, and extension at 72°C for 5 s. The progress of real-time fluorescent PCR was monitored at 530 nm. To establish external standard curves for the quantification of each HAdV, genomic DNA from each strain was diluted in a 10-fold series (10^2^ to 10^7^ copies per reaction) and analyzed with the new assay. The samples that defined the standard curve were performed in triplicate and repeated twice.

**Table 4:** Primers for real-time PCR amplification of adenovirus. The accession numbers for the viral genome sequences are HAdV-C1 (AF534906), HAdV-C2 (AC_000007), HAdV-C5 (AC_000008), HAdV-C6 (FJ349096).

Virus	Primer/probe	Gene	Position in genome	Primer sequence	Amplicon (bp)
HAdV-C1	prHAdV-C1-F	hexon	19285	AGGAAATGGCTGAAGAACT	159
	prHAdV-C1-R		19443	TTTGTAAGCCATTGGCGG	
	prbHAdV-C1		19382	FAM-GACTCATGTATATGCTCAGGCTCCT-TAMRA	
HAdV-C2	prHAdV-C2-440F	hexon	19277	AGGATGAAGAAGAGGAAGAT	181
	prHAdV-C2-620R		19457	GCGTATACAGGTTTAGCTTG	
	prb-HAdV-C2-563		19402	FAM- AAAGCGGGCTACAAATAGGAT-TAMRA	
HAdV-C5	prHAdV-C5.HEX.19261F	hexon	19261	GCTCTTGAAATAAACCTAGAAGAA	273
	prHAdV-C5.HEX.19533R		19533	TTTCATTGGGGTAGTCTTT	
	prbHAdV-C5.4		19312	FAM-GACGAGCAAGCTGAGCAGC-TAMRA	
HAdV-C6	prHAdV-C6-F	hexon	19259	CTGCACAAGTGGATGCT	162
	prHAdV-C6-R		19420	CGTCGGCAGTTCCTATTT	
	prbHAdV-C6		19343	FAM-AAACCCATGTATATGCCCAGG-TAMRA	
